# Deep learning for adaptive chemotherapy: A DDPG-based approach to optimizing tumor-immune dynamics

**DOI:** 10.1371/journal.pone.0345877

**Published:** 2026-04-08

**Authors:** Wenlang Zhu, Mingliu Zhu, Weiye Wang, Liang Xu, Jie Wu, Weiping Li, Ruru Ma, Lingli Cao

**Affiliations:** 1 Department of Gastrointestinal Surgery, The First People’s Hospital of Taicang City, Taicang Affiliated Hospital of Soochow University, Taicang, Jiangsu, China; 2 School of Mathematical Sciences, Suzhou University of Science and Technology, Suzhou, China; 3 School of Artificial Intelligence and Computer Science, Jiangnan University, Wuxi, China; 4 Department of Obstetrics and Gynecology, The First People’s Hospital of Taicang City, Taicang Affiliated Hospital of Soochow University, Taicang, Jiangsu, China; Vellore Institute of Technology, INDIA

## Abstract

In this article, we propose a deep reinforcement learning based chemotherapy regulation framework to realize personalized and dynamic optimization of cancer treatment. We use a nonlinear dynamic system to model the dynamic evolution of the tumor microenvironment including tumor cell, normal cell and immune cell interactions, with drug concentration serving as the control input variable. The Deep Deterministic Policy Gradient (DDPG) algorithm makes agents can study optimal dosing strategy in a continuous space of movement to inhibit tumor growth effectively and minimize damage to normal tissues. To make the strategy more stable, Gauss noise is added to the model to simulate physiological oscillations and uncertainties in the treatment reaction. Experimental results show that it can control the growth of tumor in various initial scenarios and the accumulation of the drug concentration with high flexibility and safety. Our technique provides a feasible technical way for precision, low toxicity adaptive chemotherapy.

## 1. Introduction

Cancer rates are on the rise across the globe-a trend that shows no signs of slowing [1]. As we observed in the World Health Organization (WHO) report, in 2020, we find that there are about 19.3 million new cancer cases and 10 million deaths globally [2]. Projections paint an even starker picture: by 2050, they could increase to 35.3 million new cases and 18.5 million deaths and be the second leading cause of death in the world, as well as cardiovascular disease [3]. Many common high-cidence cancers including lung, breast, colorectal, liver, and so on, kill thousands of patients and impair their quality of life [4]. From explore what causes the disease to how to mitigate its harms, from prevention to treatment, cancer may face a complicated list of interconnected challenges. At the heart of cancer treatment today is: how to stop tumor growth and to save healthy tissues as much as possible. Current chemotherapy seems to be shortcoming. Since they lack personalisation and have the ability to adapt dynamically to a patient’s state of change, they offer only limited success and suffer from harsh systemic side effects [5,6]. The reason for these problems lies in their one-size-fits-all nature-static protocols without taking into account how tumors grow in time, the unique differences between patients, or how a person reacts in real time. The consequences are all too real: patients suffer toxic side effects, drug resistance, and cancer relapse, hence more smarter, more adaptive and personalized treatments are needed.

To tackle this problems, mathematical modeling of tumor dynamics-a combination of biology, mathematics, and computational science-has been used to uncover cancer progression, study tumor-microenvironment crosstalk and assess treatment responses [5,7]. Models in the early part relied on simple ODE-based models like exponential or logistic growth models to track changes in tumor volume during time [8–12]. Though these models provide important base knowledge, they neglect important biological issues such as immune monitoring, spatial heterogeneity and random cell behavior. Nowadays, modeling approaches are rapidly moving towards more realistic and biologically realistic models such as Partial Differential Equation (PDE) models modeling spatial tumor growth and nutrient diffusion, stochastic process-based models modeling random mutations and environmental changes [13–15], multi-scale models modeling dynamics at molecular, cellular and tissue level [16–19], and agent-based simulations describing autonomous cell behaviors and emergent population patterns [20–23].Such models are better able to represent tumor diversity, microenvironment interactions, and adaptive resistance-thus making them important for silico experimentation and therapeutic design.

Even with these strides in modeling accuracy, a critical gap persists: translating complex tumor dynamics into actionable, personalized, and adaptive treatment strategies–the very cornerstone of precision medicine. Accurate models alone aren’t enough; crafting drug administration plans that are both globally optimal and clinically feasible remains a significant challenge. Conventional chemotherapy relies heavily on periodic bolus dosing–operationally convenient, yes, but lacking any feedback mechanism to adapt to real-time changes in disease progression [24,25]. Clinical and experimental data make clear that tumor cells often bounce back rapidly and proliferate during treatment-free windows, eroding long-term disease control. This pattern stems from a fundamental misalignment: static treatment schedules pitted against the highly nonlinear, ever-changing behavior of tumor systems. Classical optimal control theory does offer mathematical tools to derive idealized treatment plans, but these approaches come with steep tradeoffs. They demand full visibility into the system, precise knowledge of all parameters, and grapple with the “curse of dimensionality”-all factors that limit their practical use in real-world clinical settings.

Artificial intelligence (AI) is advancing at a breakneck pace [26–28], and nowhere is this more transformative than in oncology, where Machine Learning (ML) has demonstrated its value across sectors, especially in healthcare [29]. Furthermore, Deep Reinforcement Learning (DRL) is driving a paradigm shift: from simply describing tumor behavior to actively, adaptively controlling therapeutic interventions. DRL acts as an autonomous decision-making system, learning optimal treatment policies through repeated interaction with simulated environments–making it a perfect fit for closed-loop treatment management [30]. This approach builds on predictive and optimal control theory but breaks free of many of their constraints: instead of relying on explicit system equations, it learns directly from experience. In our framework, the DRL agent operates within a patient-specific tumor model, with its state space shaped by a rich mix of clinical and molecular data streams. These include genomic profiles [31,32], digitized pathological images [33–35], and longitudinal treatment outcomes [36]-all equipping the agent to track disease progression and dynamically tweak therapeutic actions in real time. Cross-domain successes further validate the feasibility of applying this learning-based control to complex biological systems [37,38]; for instance, the Proximal Policy Optimization (PPO) algorithm has been used to stabilize intricate ecosystems like food webs [39], offering compelling proof of DRL’s potential to manage nonlinear, multi-agent dynamics even amid uncertainty.

Based on these results, we propose a novel chemotherapy control scheme using DDPG to achieve personalized drug dose control. DDPG is a deep reinforcement learning method for fine-grained control of the drug concentration of chemotherapy. Unlike random policies that recommend arbitrary doses, DDPG can produce smooth, deterministic doses that are more consistent, interpretable, and clinical-able. The learning environment is determined by a nonlinear dynamic model, which includes tumor cells and immune effector cells and normal host tissues. The model takes into account resource competition and immune activation and stochastic noises to simulate uncertainty in biological system and clinical treatment. The models are general and applied. In appropriate initial conditions, we analyze the ability of DDPG to maintain tumor suppression and to lower the total drug exposure-a metric which is essential to minimize treatment toxicity. We also test the performance of DDFG agent in realistic pathological states and demonstrate the possibility of outperforming the classical fixed-dose regimen.

The paper is organized as follows. In Section 2, the mathematical model of the tumor dynamics model and tumor, immune and normal cells relations are presented. Section 3, the main idea of the DDPG algorithm and applications to continuous drug control are described. Simulations are described in Section 4 to illustrate the control performance and the key therapeutic indicators are analyzed. Section 5 summarizes the main contributions and their impact on adaptive cancer therapy and future research directions are highlighted.

## 2. The tumor model

The model integrates core biological mechanisms within the tumor microenvironment. The simulation incorporates populations of immune cells capable of detecting the presence of tumors, proliferating in response, and actively attacking malignant cells through dynamic interactions [9]. Importantly, the emergence of a clinically detectable tumor does not indicate a complete failure of immune surveillance-even if an immune response is initiated its strength may be inadequate to suppress the rapid growth of cancer cells. From a model of ecological dynamics, the competition between normal and tumor cells for limited resources is modeled, and immune and tumor cell interactions are modeled as predator-prey interactions. This allows us to build a simulation system which accounts for the complex interplay between tumor growth and immune response.

Tumor cell growth follows a logistic pattern, approaching saturation under limited resources, and is suppressed by two main factors: specific killing by immune cells and competition with normal cells for resources. Normal cells also exhibit self-limited proliferation, but their growth is significantly inhibited by tumor invasion. The immune system is modeled as a responsive activation mechanism: its proliferation increases with tumor burden but exhibits a saturation effect, potentially leading to functional exhaustion when the tumor becomes too large; meanwhile, immune cells also undergo natural decay, further increasing the complexity of system regulation [40,41].

Based on the aforementioned biological assumptions, the system state is described by three variables: the normalized populations of normal cells *N*(*t*), tumor cells *T*(*t*), and immune cells *I*(*t*). The system dynamics are governed by the following set of coupled ordinary differential equations:


$$\left\{\dot{N}=N(1-N)-c_{4}TN,\;\dot{T}=rT(1-bT)-c_{2}IT-c_{3}TN,\;\dot{I}=1+\frac{\rho IT}{1+T}-c_{1}IT-d_{1}I,\right)$$ (1)

where *r* represents the proliferative advantage of tumor cells relative to normal cells; when *r* > 1, tumor cells proliferate more rapidly, driving faster tumor expansion. The parameter *b* reflects growth inhibition under high tumor density, capturing the saturation effect due to limited space and nutrients. A bidirectional dynamic exists between tumor and immune cells: *c*_2_ denotes the immune system’s ability to eliminate tumor cells, while *c*_1_ characterizes the suppressive effect of tumor cells on the immune system, reflecting their capacity to weaken immune attacks through immune evasion mechanisms. Competition between normal and tumor cells for limited resources in the microenvironment is governed by *c*_3_; a higher *c*_3_ value indicates stronger suppression of tumor growth by normal tissue. Conversely, *c*_4_ represents the inhibitory impact of tumor cells on normal cells, reflecting how their invasive growth disrupts the homeostasis of normal tissue. Immune cell activation increases with tumor burden, modeled by the nonlinear term $\frac{\rho IT}{1+T}$, where ρ controls the strength of the immune response; the saturation form prevents unrealistic, excessive activation at high tumor levels. Finally, *d*_1_ represents the natural decay rate of immune cells, indicating that continuous replenishment is required to sustain effective immune surveillance [9].

Such a normalized model preserves the original biological mechanisms as well as numerical stability and parameter interpretation, providing a high-quality simulation framework for developing future intelligent control strategies. It connects the classical tumor-immune dynamic models [40,42] with population ecological competition models [9], resulting in a global system which is capable of capturing complex interactions of the tumor microenvironment.

**Fig 1:**
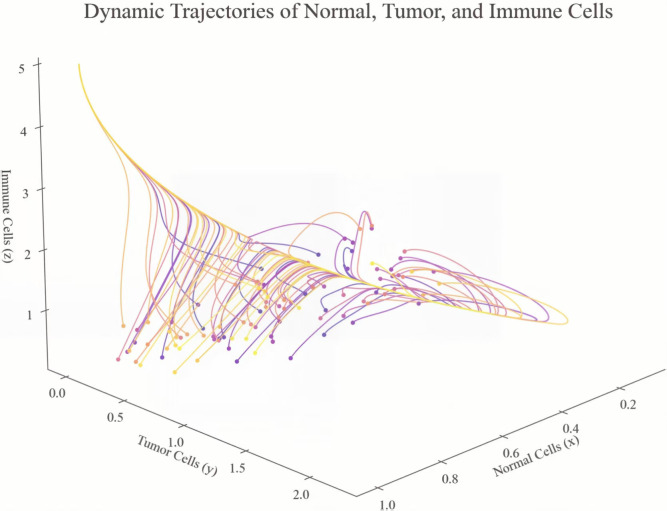
The trajectories of the dynamical tumor model with distinct initial conditions.

Fig 1 displays the temporal dynamics of the same model at 100 different starting points in order to reveal the different evolution patterns. In tumor context this capacity is tumor-dependent and associated with local physiological conditions, which generally corresponds to a number of 10^11^. Our normalization policy not only simplifies model scaling but also improves numerical robustness and allows more intuitive treatment of interactions among different cells. All further simulations involving the crosstalk between normal, tumor and immune cells follow this uniform scaling pattern and have a reliable and consistent analysis throughout the study.

To incorporate the effect of drug intervention into the model system, the following drug action function is introduced [9]:


$$f_{i}(u(t))=a_{i}\left(1-e^{-u(t)}\right),$$ (2)

where *u*(*t*) is the concentration of the drug concentration in the tumor microenvironment at time *t*, whose dynamics are determined by the given dosage rate *v*(*t*) and the clearance rate *d*_2_ in the body. The function *f*_*i*_(*u*(*t*)) is the cytotoxicity of the drug at *u*(*t*). To simplify this, exponential decay is *k* = 1, and drug sensitivity coefficients $a_{1},a_{2}$ and *a*_3_ are normal cells, tumor cells and immune cells respectively. By incorporating these drug effect terms into the original system (1), an extended dynamic model that includes therapeutic intervention is obtained:


$$\left\{\dot{N}=N(1-N)-c_{4}TN-N\cdot a_{1}(1-e^{-u(t)}),\;\dot{T}=rT(1-bT)-c_{2}IT-c_{3}TN-T\cdot a_{2}(1-e^{-u(t)}),\;\dot{I}=1+\frac{\rho IT}{1+T}-c_{1}IT-dI-I\cdot a_{3}(1-e^{-u(t)}),\;\dot{u}=v(t)-d_{2}u.\right)\;$$ (3)

For the convenience of numerical simulation and analysis, we firstly standardize the variable, *x*(*t*)=*N*(*t*) represents the normal cells population, *y*(*t*)=*T*(*t*) denotes the tumor cells population and *z*(*t*)=*I*(*t*) indicates the immune cells population. Real biological systems are inherently variable due to unpredictable physiological and environmental fluctuations. Tumor dynamics are influenced not only by internal mechanisms but also by stochastic external factors such as immune variability and metabolic heterogeneity. To account for these sources of uncertainty and enhance the model’s realism, we formulate the system as a set of Stochastic Differential Equations (SDEs). This is a standard and rigorous approach to modeling systems that exhibit intrinsic random behavior. We employ the Euler-Maruyama method for the numerical integration of this SDE system. This method correctly discretizes both the deterministic (drift) and stochastic (diffusion) components, replacing the need for separate deterministic and ad-hoc noise models. The resulting discrete-time formulation is as follows:


$$\left\{ \begin{array}{c} x_{t+1}=x_{t}+\left(\frac{dx}{dt}\right)\Delta t+\sigma_{1}\sqrt{\Delta t}\xi_{1},\\ y_{t+1}=y_{t}+\left(\frac{dy}{dt}\right)\Delta t+\sigma_{2}\sqrt{\Delta t}\xi_{2},\\ z_{t+1}=z_{t}+\left(\frac{dz}{dt}\right)\Delta t+\sigma_{3}\sqrt{\Delta t}\xi_{3},\\ u_{t+1}=u_{t}+\left(\frac{du}{dt}\right)\Delta t+\sigma_{4}\sqrt{\Delta t}\xi_{4}, \end{array}\right.$$ (4)

where $\left(\frac{dx}{dt}),(\frac{dy}{dt}),(\frac{dz}{dt}),(\frac{du}{dt}\right)$ represent the deterministic drift terms from the right-hand side of Eq. (3) (with *v*(*t*) replaced appropriately in the contex*t* of control), and Δt is the simulation time step. The terms $\sigma_{i}$ are the noise intensities. The $\xi_{i}$ are independent random variables drawn at each time step from a standard normal distribution, 𝒩(0,1). Significantly, the scaling of the stochastic term by $\sqrt{\Delta t}$ is the usual procedure to model a Wiener process step. Thus the statistical properties of the aggregate noise are consistent and independent of the chosen choice of Δt. This is a powerful model for modeling biological stochastics.

## 3. Method

Due to the huge complexity of tumor dynamics, data-driven machine learning can be quite useful. DRL is especially interesting in this sense, by coupling adaptive decision making with powerful representation capability of DL [43]. In DRL, an agent continuously interacts with its environment, and improves its behavioral policy by trial and error by reward and penalty. Deep neural networks automatically extract hierarchical features from raw high-dimensional data in DRL to efficiently sense highly complex environmental states. Through experience replay and policy gradient optimization, DRL is able to balance exploration and exploitation by mapping environmental observations to the optimal action patterns to maximize long-term cumulative rewards. This is a natural link between dynamic policy optimisation and end-to-end learning, which provides DRL with autonomous decision making capabilities in complex real-world scenarios.

**Fig 2:**
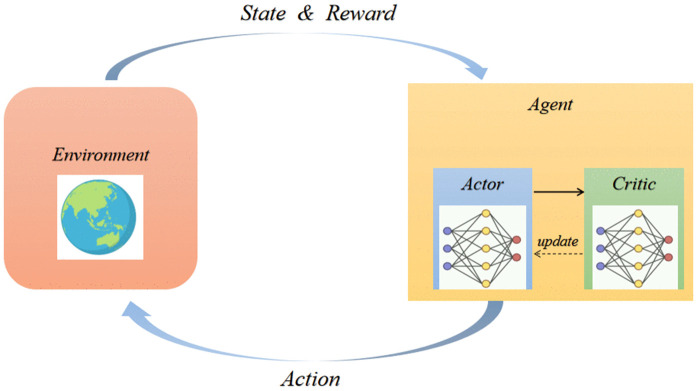
The framework of proposed DRL solution.

Fig 2 shows the interaction process between an agent and its environment in DRL. The environment gives the agent the current state and a reward signal (environment condition and feedback about the action done before), respectively. The agent consists of two components: actor and critic. The actor chooses an action based on the current condition, utilizing a policy that maps states to actions to maximize long-term rewards, performing this action in the environment and achieving new state and new reward; the critic evaluates the expected future return of the given action with respect to the policy. Depending on the critics performance, the Actor updates its policy to choose better actions in such settings; the Critic reduces the value estimation. This process of “perception-decision-execution-learning” iterates throughout and allows an agent to gradually improve its decision making with trial and error, ultimately learning to maximize the long-time rewards and achieve the task in highly complex environments.

**Fig 3:**
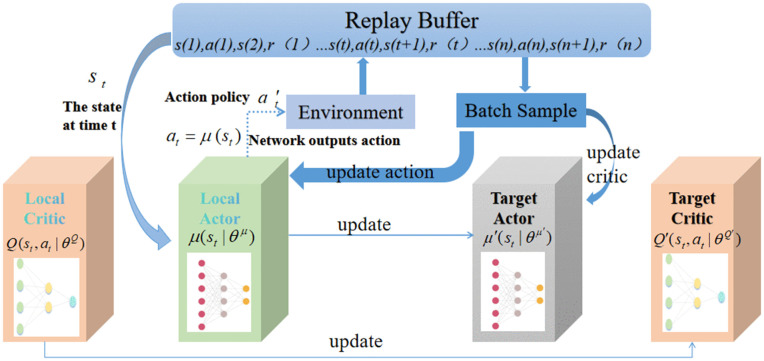
The schematic framework of DDPG algorithm.

DDPG is a well-known DRL algorithm [44], widely recognized for its excellent performance in continuous control tasks, offering stable convergence and the ability to effectively handle high-dimensional action spaces. An off-policy Actor-Critic method, DDPG significantly improves sample efficiency and training stability using experience replay and target networks. In this paper, DDPG is utilized because it learns deterministic policies that is needed in applications that require a precise and consistent control decision. DDPG uses the powerful feature extraction power of deep neural networks coupled with the principled approach of Q-learning, which extracts important information from high-dimensional inputs and improves long-term cumulative rewards by iterative policy optimization. Fig 3 illustrates the schematic framework of the DDPG algorithm.

As shown in Fig 3, during the environmental interaction and experience collection stage, the agent has interacted with the environment in real time through the local actor. Specifically, the agent has first observed the environment to obtain the current state *s*_*t*_. The local actor has then used its parameterized deterministic policy function $\mu (s_{t}|\theta^{\mu })$ to directly compute and output the corresponding action *a*_*t*_. Upon execution of this action in the environment, the system has transitioned to the next state *s*_*t*+1_ and received an immediate reward *r*_*t*_. To break temporal correlations among data samples and enable off-policy learning, the resulting transition tuple ($s_{t},a_{t},r_{t},s_{t+1}$) has been stored in the replay buffer.

To ensure training stability, DDPG has incorporated a batch sampling mechanism. The algorithm has randomly drawn a mini-batch of historical transition tuples from the replay buffer and fed it into the local critic. The primary role of the local critic has been to evaluate the quality of the current policy by estimating the state-action value $\mu (s_{t},a_{t}|\theta^{\mu })$, which denotes the policy rather than the value function–thereby effectively “scoring” the actions generated by the actor. Simultaneously, the algorithm has maintained a set of target networks (target actor and target critic). These target networks have been used to compute stable target *Q*-values, which serve as a consistent learning objective for updating the local critic. This design has significantly mitigated training instability and reduced oscillations commonly observed in DRL with neural function approximators.

During learning, the algorithm has concurrently updated the local actor and local critic using sampled mini-batch data. The local critic has refined its accuracy by minimizing the mean squared error between predicted and target *Q*-values, while the local actor has updated its policy parameters along the gradient provided by the critic–specifically, the policy gradient derived from the critic’s value estimates–to favor actions with higher expected returns.

*Remark.* In our study, the variables involved in the tumor model are constrained within fixed and reasonable ranges. As a result, we did not encounter obvious hyperparameter sensitivity or Q-value overestimation bias during training.

The Actor network aims to maximize the long-term cumulative reward as estimated by the Critic network. Its parameters $\theta^{\mu }$ are updated using gradient ascent based on the policy gradient method. The Critic network evaluates the value of the current state-action pair using the value function $Q(s_{t},a_{t}|\theta^{Q})$, where $\theta^{Q}$ represents the parameters of the Critic network. The learning objective of the Critic is to minimize the mean squared error (MSE) between its predicted value and the target value. The loss function is defined as [44]:


$$\begin{array}{c} L(\theta^{Q})=𝔼\left[(y-Q(s_{t},a_{t}|\theta^{Q}){)}^{2}\right], \end{array}$$ (5)

where the target value is given by $y=r_{t}+\gamma Q^{\prime }(s_{t+1},\mu^{\prime }(s_{t+1}|\theta^{\mu^{\prime }})|\theta^{Q^{\prime }})$, γ is the discount factor, and *Q*’ and $\mu^{\prime }$ denote the outputs of the target Critic and target Actor networks, respectively.

For further training stability, DDPG proposes target network. The parameters of the target Actor network $\mu^{\prime }(s_{t}|\theta^{\mu^{\prime }})$ and the target Critic network $Q^{\prime }(s_{t},a_{t}|\theta^{Q^{\prime }})$ are updated by slowly tracking the parameters of their corresponding local networks through a soft update mechanism, defined as:


$$\begin{array}{c} \theta^{\mu^{\prime }}\leftarrow \tau \theta^{\mu }+(1-\tau )\theta^{\mu^{\prime }},\\ \theta^{Q^{\prime }}\leftarrow \tau \theta^{Q}+(1-\tau )\theta^{Q^{\prime }}, \end{array}$$ (6)

where τ is a small positive constant that regulates the update rate. This stabilizes the learning by providing more stable target values for policy and value updates. Hence, DDPG can learn effectively and optimize policies in continuous action spaces and gradually improve the agent’s decision making power in complex situations.

In this experiment, we use the DDPG algorithm to automatically control the dynamics model of the tumor and to generate a time-dependent drug concentration profile that suppresses tumor growth. Contrary to popular empirical control methods such as fixed dose or periodic drug doses, DDPG can generate a continuous and fine-grained action output in real time according to the current tumor state and map them into personalized drug concentration actions. Based on this approach, the tumor model described by Eq. (3) is transformed into the following form:


$$\left\{\dot{x}=x(1-x)-c_{4}\;xy-x\cdot a_{1}(1-e^{-u(t)}),\;\dot{y}=ry(1-by)-c_{2}\;yz-c_{3}\;xy-y\cdot a_{2}(1-e^{-u(t)}),\;\dot{z}=1+\frac{\rho \;yz}{1+y}-c_{1}\;yz-d\;z-z\cdot a_{3}(1-e^{-u(t)}),\;\dot{u}=a_{t}-d_{2}u,\right)\;$$ (7)

where *a*_*t*_ is the control action of the DDPG algorithm at current time-step *t*. Different from *v*(*t*), the decision variable is replaced by *a*_*t*_, which represents a transition from fixed-dose controlled to dynamic, state-feedback controlled control. The DDPG algorithm employs an actor-critic network architecture to learn an optimal drug dosage policy in a continuous action space, predicting the best drug dosage on the current time step of the system in order to adjust for personalized, time-dependent regulation. As the agent is trained, it continuously adapts to long-term target –by effectively keeping the tumor growing without excessive medication. This adaptive mechanism can lead to better treatment performance, less side effects, and a safer and more accurate treatment.

Training DRL agents in environments requires correct perception of the environment and thus the decision making itself depends critically on the observation space. To fully capture system dynamics and guide the agent in making rational therapeutic decisions, constructing an informative and representative observation variable *O*_*t*_ is of great importance. In this study, *O*_*t*_ simply adds the number of three specific types of cells (tumor cells, immune cells, normal cells) at current time step t and their time derivatives (or of current state and rate of change). By taking into account static state information and dynamical changes *O*_*t*_ brings the agent deeper environmental information into more insight regarding complex biological processes and may lead to more accurate and timely drug decisions. The design of *O*_*t*_ is as follows:


$$\begin{array}{c} O_{t}=[\begin{array}{cccccc} x_{t}, & y_{t}, & z_{t}, & {\dot{x}}_{t}, & {\dot{y}}_{t}, & {\dot{z}}_{t} \end{array}{]}^{T}, \end{array}$$ (8)

where $x_{t},y_{t},z_{t}$ and ${\dot{x}}_{t},{\dot{y}}_{t},{\dot{z}}_{t}$ represent the quantities and rates of change, respectively, for the three types of cells at the current time step. Observing the current state on the position of the current condition may not capture the current states evolution and possible evolution. In dynamic conditions, such as biological processes, control systems or complex decisions, a state’s future behavior may depend not only on its current state, but also on dynamic features such as the change rate. Therefore, in addition to observing the current situation we consider the time derivatives of the variables, building a multidimensional observation space combining both “position” and “moment”. Such a rich representation makes the current position as well as the information density of the observations much more complete and allow the agent to predict future status, compare the control results and make more forward decisions.

As illustrated in the working principle of the DDPG algorithm in Fig 3, the reward function plays a central role in the training of the reinforcement learning agent. It serves not only as the “intrinsic motivation” driving the agent’s exploration and learning but also directly determines the direction and final performance of policy optimization. In this study, the experimental objective is to effectively suppress tumor growth while maximally preserving normal tissue cells and avoiding excessive damage. To this end, the design of the reward function is closely aligned with this dual objective, focusing on the extent of tumor cell reduction and the maintenance of normal cell levels. The specific formulation is as follows:


$$reward=\sum_{t=1}^{T}r_{t},$$ (9)

where $r_{t}=-e_{y}+e_{x}-\sqrt{e_{y}}+\sqrt{e_{x}}$, $e_{y}=y_{t}-0$ across all dimensions at step *t*, $e_{x}=x_{t}-0$ across all dimensions at step *t*. The total reward over the entire process is ob*t*ained by summing *r*_*t*_ over all time steps. The reward for all time steps is simply summing over all times*t*eps. Firstly, the term $-e_{y}$ penalize the tumor cell count, and higher tumor burden results in a harder punishment, which creates a fast, direct effect signal on high tumor cell counts. As the tumor cell population approaches zero, the gradient signal generated by the linear error term becomes vanishingly weak, often causing the agent to lose the “motivation” required to eliminate the final residual cancer cells. To mitigate this issue, a square-root term is introduced to non-linearly amplify the error. Mathematically, as the error *e*_*y*_ tends toward zero, its derivative approaches infinity:


$$\lim_{e_{y}\to 0^{+}}\frac{d}{de_{y}}\sqrt{e_{y}}=\lim_{e_{y}\to 0^{+}}\frac{1}{2\sqrt{e_{y}}}=+\infty .$$ (10)

Consequently, even when the residual tumor burden is minimal, the agent still receives a robust penalty signal, ensuring the thorough eradication of malignant cells. Secondly, for normal cells +*e*_*x*_ provides a linear positive incentive, encouraging the maintenance of a high cell level and the biological principle of diminishing marginal utility is integrated into the reward structure. Specifically, for lower normal cell populations *e*_*x*_, small increments in cell counts yield substantial positive reinforcement. This is mathematically justified by the derivative of the square-root incentive term:


$$\frac{d}{de_{x}}\sqrt{e_{x}}=\frac{1}{2\sqrt{e_{x}}}.$$ (11)

As $e_{x}\to 0^{+}$, the gradient approaches infinity, providing a powerful learning signal for the agent to avoid lethal toxicity. As *e*_*x*_ increases, the marginal reward decreases, preventing the agent from over-prioritizing normal cell growth over necessary tumor suppression, and when the cell count is well-heaped, the returns are diminished, i.e., the agent does not have to resort to excessively aggressive strategies. Finally, unlike traditional methods, this framework does not explicitly incorporate a penalty term for drug concentration in the reward function, but instead achieves implicit equilibrium through system dynamics. Under this mechanism, if the gain in tumor reduction from increasing the dosage is outweighed by the loss incurred from damage to normal cells, the total reward *r*_*t*_ will decrease. Consequently, the DDPG agent spontaneously seeks the minimum effective dose required to achieve tumor reduction goals, thereby effectively constraining the drug concentration without the need for an explicit penalty. The reward function employs linear terms to provide fundamental reward and penalty signals, while utilizing square root terms (nonlinear terms) to regulate sensitivity and stability. Rather than directly penalizing the numerical value of the drug concentration, it constrains drug administration through the consequences of drug toxicity on normal cells, thereby maximizing tumor suppression while ensuring that normal tissue survival rates are maintained at a safe level, not only effectively changing challenging control goals in a way that provides good reinforcement learning signals, but also motivating the agent to discover efficient and robust control strategies in highly dynamic environments.

Algorithm 1 presents the tumor treatment control framework based on the DDPG algorithm. This method employs an Actor-Critic architecture with target networks and experience replay, enabling efficient learning of optimal drug administration policies in continuous action spaces. By designing a reward function for tumor suppression and normal cells preservation, the agent constantly changes the strategy based on interaction with the environment, achieving exact control of tumor growth. This scheme is able to cope with the nonlinear dynamics of the tumor system and is good to obtain stable convergence and robustness. Experimental results and performance are provided in Section 4 to confirm the effectiveness of this treatment and potential application to personalized treatment design.

**Algorithm 1** The simplified DDPG in dynamical tumor system

1: Initialize actor $\mu_{\theta }$, critic $Q_{\phi }$, target networks $\mu_{\theta^{\prime }},Q_{\phi^{\prime }}$, replay buffer ℬ

2: Set discount γ, batch size *M*, soft update rate τ, noise process 𝒩

3: **for** each episode **do**

4:  Observe initial state *s*_0_, reset noise

5:  **for** *t* = 0 to T−1 **do**

6:   Select action: $a_{t}=\mu_{\theta }(s_{t})+{𝒩}_{t}$

7:   Execute *a*_*t*_, get reward *r*_*t*_ and next state *s*_*t*+1_

8:   Store $\left(s_{t},a_{t},r_{t},s_{t+1}\right)$ in ℬ

9:   Sample minibatch 𝒟 from ℬ

10:   Compute target: $y_{i}=r_{i}+\gamma Q_{\phi^{\prime }}(s_{i+1},\mu_{\theta^{\prime }}(s_{i+1}))$

11:   Update critic: $\phi \leftarrow \phi -\alpha_{\phi }\nabla_{\phi }ℒ$, where $ℒ=\frac{1}{M}\sum (y_{i}-Q_{\phi }(s_{i},a_{i}){)}^{2}$

12:   Update actor: $\theta \leftarrow \theta +\alpha_{\theta }\nabla_{\theta }Q_{\phi }(s,\mu_{\theta }(s))$

13:   Soft-update target networks: $\theta^{\prime }\leftarrow \tau \theta +(1-\tau )\theta^{\prime }$, $\phi^{\prime }\leftarrow \tau \phi +(1-\tau )\phi^{\prime }$

14:   $s_{t}\leftarrow s_{t+1}$

15:   **if** *s*_*t*+1_ is terminal **then break**

16:   **end if**

17:  **end For**

18: **end For**

## 4. Experimental results

All programs in this study were implemented in Python 3 using the PyTorch framework for building and training deep reinforcement learning models. In order to precisely control tumor growth, we employed DDPG, suitable for continuous action spaces and for learning fine-grained drug dosing strategies that meet practical requirements. In the training step, the agent interacts with the environment to collect experience which is stored in a replay buffer. Mini-batches sampled from this buffer are used to update the network parameters repeatedly. As large matrix operations and gradient calculations are performed, we exploit GPU acceleration to improve training efficiency and convergence speed. Additionally, to avoid severe tumour growth or damage to normal cells due to sub-optimal actions during the early exploration, we present a state-aware function: if the tumor counts are greater than a given safety threshold or the normal cell counts fall below a critical value, this episode is dropped immediately, as learning and computation cost do not work.

To ensure the reproducibility of our study, we detail the key hyperparameters used for our DDPG agent. The actor and critic networks both utilized an MLP architecture with two hidden layers of 256 units each ([256, 256]). The learning rate was set to 0.001 for both networks. We employed a replay buffer with a capacity of 1,000,000 samples and used a batch size of 256 for each training update. The discount factor (γ) was 0.99, and the soft update coefficient for the target networks (τ) was 0.005. We outline below the key parameters and setup details, which are determined by referencing the work of Pillis [9]:


$$\begin{array}{cccccccc} \rho & =0.01, & c_{1} & =0.3, & c_{2} & =0.165, & c_{3} & =1.0,\\ c_{4} & =0.3, & d & =0.2, & d_{2} & =1.0, & r & =1.5,\\ b & =0.3, & a_{1} & =0.2, & a_{2} & =0.3, & a_{3} & =0.1. \end{array}$$

### 4.1. Sensitivity to Initial Conditions and Treatment Duration

**Fig 4:**
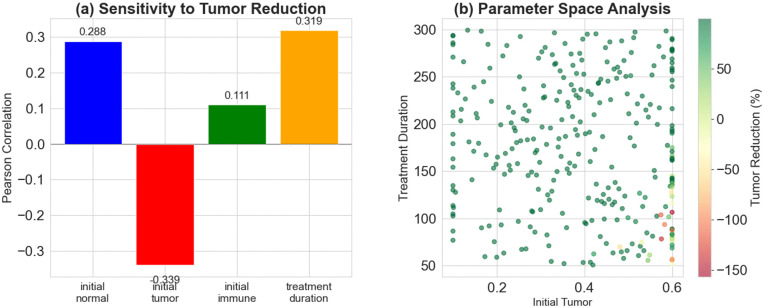
Monte Carlo sensitivity assessment of the DDPG-based control model. **(a)** Sensitivity ranking determined by calculating the Pearson correlation coefficients between each parameter and the tumor shrinkage rate; **(b)** Analysis of the synergistic effect between the two key parameters–initial tumor burden and treatment duration.

In order to assess the effect of uncertainty in the initial state on the patient’s treatment, we performed a global sensitivity test to assess robustness of the proposed control. We generated 300 representative sample sets by Monte Carlo-based approach with Latin Hypercube Sampling (LHS), and systematically evaluated four key uncertain factors(3 initial values and the treatment time): initial normal cell concentration (0.5–0.9), initial tumor cell concentration (0.1–0.6), initial immune cell concentration (0.05–0.2) and total treatment time (50–300 time steps). For each parameter set, we pre-trained the DDPG policy, and recorded the important performance indicators–tumor volume reduction rate and total cumulative drug dosage–to evaluate the performance of the controller under various biologically realistic scenarios. Results are presented in Fig 4.

Fig 4(a) is shown that the concentrations of initial immune cells and normal cells are significantly positively correlated to treatment efficacy, highlighting the key role of immune status and tissue health in tumor control: higher initial immune activity increases treatment response, while sufficient normal cell reserves may reduce systemic damage during therapy. In addition, longer treatment times lead to higher tumor suppression rates, emphasising the importance of enough intervention time to achieve deep remission. However, more initial tumor burden makes it harder to reach the desired control goal, leading to lower tumor shrinkage rates, and suggesting that a late intervention is needed. As we conclude from all results, initial tumor cell count and treatment duration are the two most sensitive variables, whose their variations have a decisive impact on the final outcome.Further extreme scenario analysis (as shown in Fig 4(b)) focuses on the boundary combinations of these two sensitive parameters. The results indicate that control failure–defined as the inability to effectively suppress tumor growth–occurs only under the most extreme conditions, specifically when the treatment duration is very short (close to 50 steps) and the initial tumor burden is very high (close to 0.6). In the vast majority of other parameter configurations, the proposed control strategy achieves stable tumor suppression even under significant parameter variations, demonstrating strong robustness and broad adaptability.

**Fig 5:**
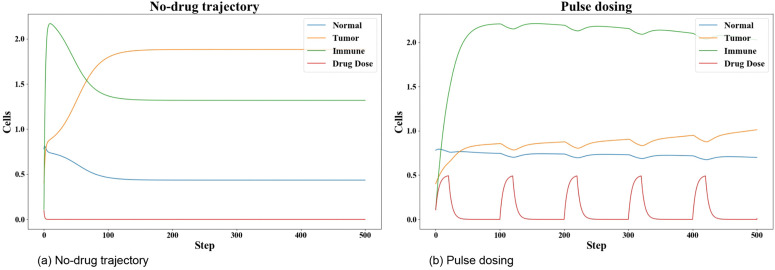
Baseline comparison of the revised tumor model dynamics. (a) illustrates the system trajectories in a drug-free scenario. (b) shows the system response to a standard periodic pulse dosing regimen.

We first analyzed the dynamics of our revised tumor model under two fundamental scenarios: (1) no therapeutic intervention, and (2) a standard, simple treatment regimen. This analysis is necessary in order to justify the need for an advanced control strategy, such as DDPG. The results are presented in Fig 5.

As shown in Fig 5(a), in the absence of any drug intervention, the tumor population (orange line) grows significantly and stabilizes at a high, persistent level, while the normal cell population (blue line) is suppressed. This result confirms the model’s baseline behavior and clearly demonstrates that therapeutic intervention is necessary.

In Fig 5(b), we implemented a standard pulse dosing regimen. This analysis shows that while the periodic drug application (red line) successfully suppresses the tumor to a lower level than the no-drug case, it is insufficient to eliminate the tumor. The tumor population persists in an oscillatory state and never reaches zero. These two baseline scenarios confirm the need for treatment and demonstrate the limitations of a simple, standard dosing protocol. This provides the necessary context for evaluating the performance of our proposed DDPG agent in the following sections.

### 4.2 Simulation of tumor treatment drug regulation using the DDPG Algorithm

For the study of tumor-immune dynamics in immunosuppressive conditions, we set the initial immune cell level to *I*(0)= 0.10 and model a significantly weaker immune response, for which we can compare the treatment performance in a more relevant, immune-compromised setting. All cell populations were normalized such that they were kept constant and equally distributed within the simulation. We also imposed a physiological viability constraint: the fraction of normal cells must be higher than 0.7 during the simulations and preserve essential tissue function to provide biologically plausible results. The tumor system was initialized with *N*(0) = 0.78 (normal cells), *T*(0) = 0.35 (tumor cells), and *I*(0)= 0.10 (immune cells). It is important to clarify that the legend labels in the figures depict the system’s evolution after treatment initiation, rather than the initial conditions. Hence, any apparent mismatch between the starting values and the plotted trajectories is expected and reflects the dynamic response to therapeutic intervention.

On a real biological systems, tumor dynamics is not only affected by immune changes but also by external influences, such as environmental changes, measurement noise, and individuals-by-individual physiological heterogeneity. To account for these sources of uncertainty and enhance the ecological validity of our simulation framework, additive stochastic noise was incorporated into the state transition process, as specified in Eq. (4). In particular, a Gaussian noise of zero mean and unit standard deviation was added at every time step to distort the state and expose the agent to more realistic variability during training. This can be used to develop more robust control policies that are less sensitive to transient disturbances and that can perform at noisy and non-stationary levels.

**Fig 6:**
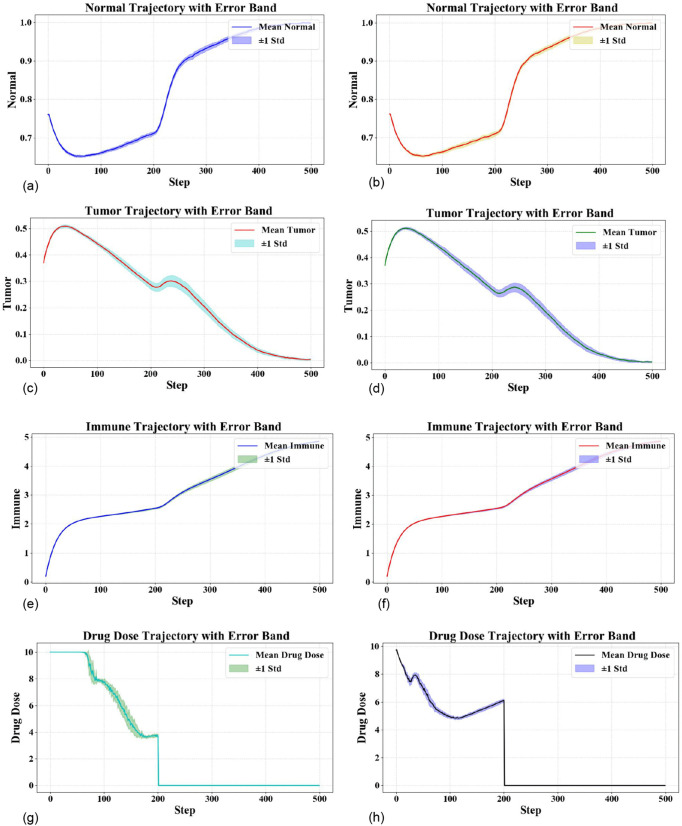
Trajectories behavior in a noisy setting Comparison between a model trained in noise-free conditions (left) and one trained with added noise (right).

**Fig 7:**
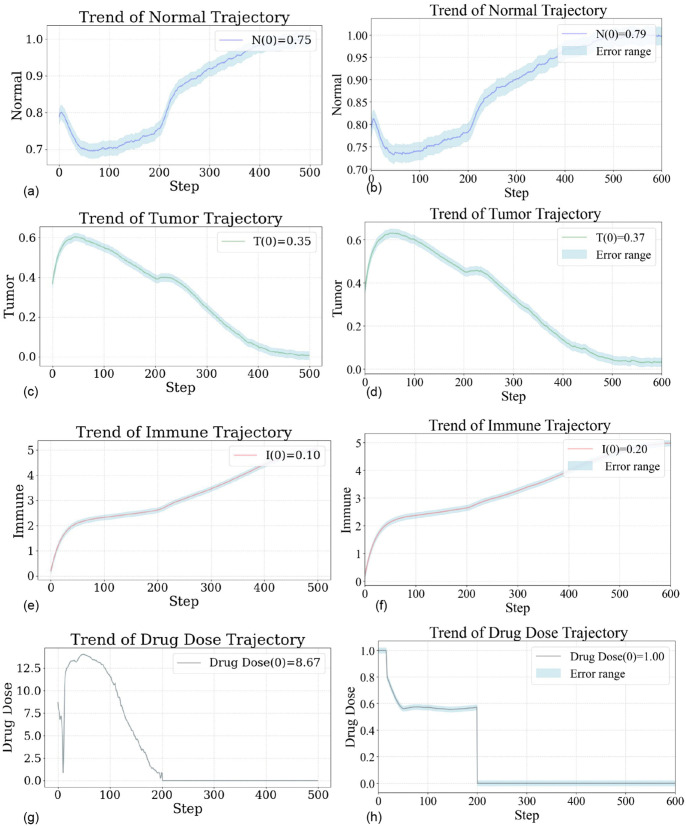
Control performance comparison before and after reward function optimization. (left) Model with original reward function; (right) Model with optimized reward function.

Here, we have compared our two models, one trained in noise-free conditions, and the other trained in noisy-augmented conditions, under the same starting conditions as mentioned before. All tests were performed in a dynamic environment with added Gaussian white noise to simulate the inevitable stochastic perturbations present in real physiological systems. For all scenarios presented in Figs 6 and 7, we imposed a pre-defined experimental constraint: all drug administration is stopped at *t* = 200. This design choice was made to test our hypothesis that an initial treatment phase is sufficient to allow the immune system to recover and autonomously suppress the remaining tumor, thereby reducing total drug exposure and toxicity. Fig 6 shows the dynamic evolution trajectories of normal cells, tumor cells, immune cells, and drug concentration. The left column displays the performance of the model trained without noise when tested under noisy conditions, while the right column presents the response of the model trained with noise injection when evaluated in the same noisy testing environment.

It is clear from our experiment that for noisy conditions, the trained model without noise suppression has a lower control than the trained noise augmentation model. In particular, the noisy-free trained model does not be robust enough for normal cells. In early treatment, the model does fail to respond to system perturbations, leading to a smaller fraction of normal cells, and the smallest is smaller than the noise-trained model. We conclude that the nonadaptive training during noise makes the controller sluggish to respond when the state changes, and is not able to adapt drug management in a timely way to mitigate damage to healthy tissue. However, in comparison to the trained models, while they show the same macro-level behavior of tumor and immune cells evolution, the control accuracy and stability are still significantly different. Moreover, by drugdose strategy, the proposed model with noise injection has a clear advantage: it achieves effective suppression of tumor cells at a lower average drug dosage, highlighting its superior control robustness.

In order to better quantify and compare the control performance of the two models we gave the Mean Absolute Error (MAE) and Relative Mean Absolute Error (RMAE) as the metrics of a measure for their control performance. These metrics effectively reflect both the convergence behavior of the model and the proximity of its output trajectory to the ideal therapeutic trajectory: lower values indicate that the control policy generated by the model is closer to the desired treatment objective–namely, achieving effective tumor suppression while minimizing drug-related side effects.

As shown in Table 1, DDPG algorithm has yielded relatively lower values for both MAE and RMAE compared to other methods. This result suggests that, under the current experimental setup, DDPG has demonstrated favorable convergence properties and has exhibited reasonably effective tumor suppression in simulated treatment scenarios, reflecting a certain degree of synergistic optimization between dosing precision and therapeutic efficacy achieved by the proposed approach.

**Table 1:** The MAE and RMAE metrics of trained models for different cell types and drug concentration.

	Gaussian noise-based training	Training without noise
**MAE in Tumor Cells**	**0.327127**	**0.334985**
**RMAE in Tumor Cells**	**0.156695**	**0.158642**
MAE in Normal Cells	0.896294	0.872042
RMAE in Normal Cells	0.806462	0.770550
MAE in Immune Cells	2.771159	3.285717
RMAE in Immune Cells	7.914898	11.866399
**MAE in Drug Concentration**	**0.174462**	**0.223684**
**RMAE in Drug Concentration**	**0.099288**	**0.159607**

These metrics are calculated as follows. The MAE is defined as:


$$\text{MAE}=\frac{1}{T}\sum_{t=1}^{T}|y_{t}-{\hat{y}}_{t}|,$$ (12)

where *T* is the total number of simulation time steps, *y*_*t*_ is the actual value of the variable (e.g., tumor cell count) at time *t*, and ${\hat{y}}_{t}$ is the desired target value. For our control objectives of tumor cells and drug concentration, the target ${\hat{y}}_{t}$ is 0.

To compare control performance across variables with different scales (e.g., cell counts vs. drug concentration), we also use the RMAE, which normalizes the MAE by the variable’s average magnitude:


$$\text{RMAE}=\frac{\sum_{t=1}^{T}|y_{t}-{\hat{y}}_{t}|}{\sum_{t=1}^{T}|y_{t}|}=\frac{\text{MAE}}{\frac{1}{T}\sum_{t=1}^{T}|y_{t}|}.$$ (13)

This formulation provides a robust, dimensionless percentage that avoids division-by-zero errors as *y*_*t*_ approaches the target of 0.

Table 1 summarizes the error values of cell type and drug concentration for the two training methods. To motivate the key control goal, we bold the contents of tumor cells and drug concentrations.

The table shows that the model with noise increase provides better control of tumor cells: its MAE and RMAE are smaller than the model without noise, indicating a better control control of the growth with low fluctuations and thus more robust. Moreover, the model is able to keep more overall normal cells of the treatment, it keeps healthy tissue. In drug dosing, the noise increase model can suppress tumor cells at smaller average drug concentration, in order to perform better in both control and dosing. Overall, noise increases the control performance of the model and its clinical applicability in more complex and dynamic conditions.

### 4.3. Comparison and analysis of control performance after reward function optimization

In this experiment, we adjusted and improved the original reward function. Instead of using the linear term of the position error as the feedback signal, the updated reward function uses a square root term of position error to make the agent more sensitive in small errors. This provides better gradient information with respect to the target state to encourage more accurate fine-tuning control. This non-linearly amplifies reward gradient for smaller errors, can make the model reach the desired value for later control and stay closer to the desired target thereby improving control accuracy and stability.

Fig 7 compares the model’s control performance with and without the reward function optimization under the same starting point and noisy conditions. As noted from dynamic response curves of the system, the model with higher reward function performs better in several ways. First, the optimized model maintains the normal cells and the minimum number of cells during treatment is much larger than for the original model, showing a more cautious regulation of drug toxicity and less off-target damage to healthy tissues.

Second, with regard to control efficiency, although both models effectively suppress tumor cells, the drug dose generated by the new model is smoother, with lower peak values and smaller total drug dose. This indicates that the new is able to achieve similar or better tumor control with less drug dose, which may have less side effect and burden for patients.

This performance is further improved by introducing the square root term into the reward function, which is nonlinear and makes the system more sensitive to small deviations.

### 4.4. Comparison of DDPG with Other Administration Schemes

In this study, we have designed and conducted a series of comparative experiments based on DDPG algorithm to systematically evaluate its performance in tumor treatment control tasks. To comprehensively assess the effectiveness of DDPG, we have employed fixed-dose drug administration, as well as the Synchronous Advantage Actor-Critic (A2C), PPO, and Soft Actor-Critic (SAC) algorithms, as benchmark methods for a thorough performance comparison against DDPG.

**Fig 8:**
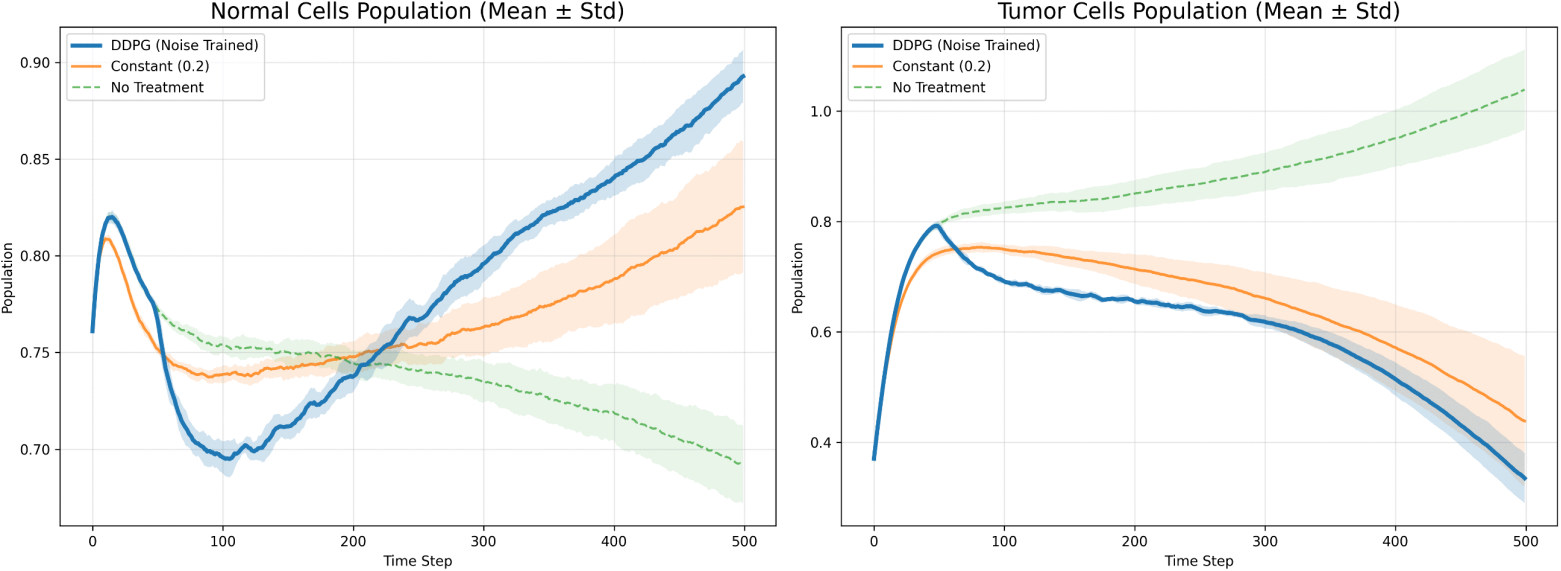
Comparison of DDPG Algorithm and Fixed Dosing Method.

As shown in Fig 8, compared to the fixed-dose strategy, the DDPG algorithm has demonstrated superior performance in tumor suppression–achieving both stronger inhibitory effects and faster onset of action. Moreover, DDPG has also outperformed fixed-dose control in protecting and restoring normal cells, promoting a more rapid and pronounced recovery in normal cell populations. The experimental results have shown that DDPG not only effectively suppresses tumor growth but also significantly enhances the long-term survival rate of normal cells, highlighting its ability to strike a favorable balance between therapeutic efficacy and tissue safety.

**Fig 9:**
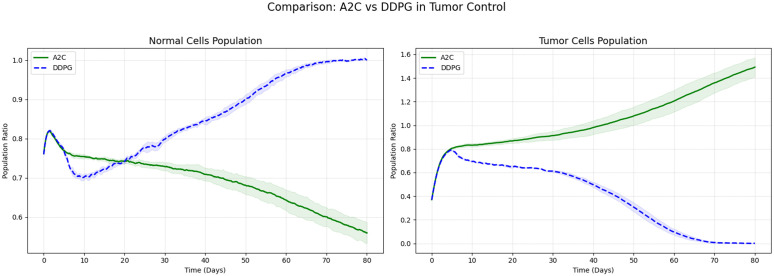
Comparison of DDPG Algorithm and A2C Algorithm.

**Fig 10:**
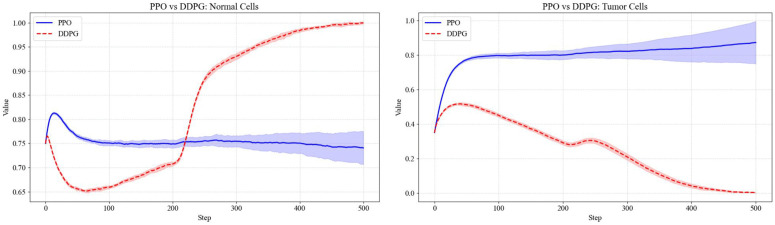
Comparison of DPPG algorithm and PPO algorithm.

In comparisons with other algorithms, DDPG has demonstrated superior tumor cell suppression capability. Specifically, DDPG has significantly outperformed A2C and PPO. As shown in Figs 9 and 10, A2C has failed to converge in this continuous control task, resulting in uncontrolled tumor cell proliferation. Although PPO has partially slowed tumor growth, it has not effectively reduced tumor cell levels and has concurrently suppressed the recovery of normal cells. In contrast, by leveraging its effective modeling of deterministic policies in continuous action spaces, DDPG has achieved a consistent and steady decline in tumor cell proportion, demonstrating stronger control performance and therapeutic potential.

**Fig 11:**
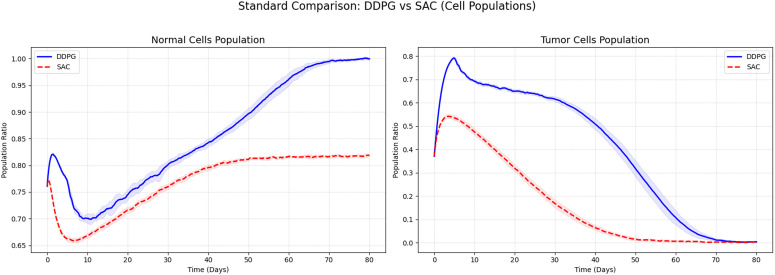
Comparison of DDPG Algorithm and SAC Algorithm.

Compared with the SAC algorithm, DDPG has achieved comparable final tumor suppression performance, yet the two methods have exhibited distinctly different learning dynamics. As shown in Fig 11, SAC has slightly outperformed DDPG in exploration efficiency and initial convergence speed, owing to its maximum entropy optimization mechanism. However, during long-term treatment, although SAC has demonstrated marginally stronger tumor cell suppression than DDPG, it has also imposed greater suppression on normal cells–reducing their proportion to below 0.7 at its lowest point–which may adversely affect tissue homeostasis. In contrast, DDPG has maintained effective tumor control while better preserving normal cells, keeping their population at a higher and safer level. Taking both therapeutic efficacy and tissue safety into account, we have concluded that DDPG demonstrates superior overall performance compared to SAC.

Finally, the numerical results in Table 2 have clearly presented a systematic comparison between DDPG and other mainstream reinforcement learning control strategies–namely SAC, PPO, and A2C. The results have shown that DDPG has achieved the lowest RMAE in the tumor cell suppression task, significantly outperforming the other methods. This metric has reflected DDPG’s more stable convergence behavior during training. Overall, DDPG has demonstrated clear advantages in both control accuracy and algorithmic stability, further validating its potential and reliability for adaptive tumor therapy.

**Table 2:** Comparison of RMAE in Tumor Cells.

	DDPG	A2C	PPO	SAC
RMAE	0.156695	0.248232	0.381268	0.317998

## 5. Conclusion and prospect

In this article, we propose an adaptive chemotherapy control method based on DDPG in order to intelligently intervene in tumor systems. Based on the combination of DDPG agent and nonlinear dynamic model of tumor-immune and normal cells, the method learns optimal drug concentration control policies in this model, overcoming the limitations of fixed-dose protocols and more personalized and dynamic adaptiveness. In simulations, we evaluated the method under the random noise introduced in experiments with DDPG. According to these in silico experiments, DDPG can generate fine-grained and time-varying dosing schedules, effectively suppressing tumor burden within the model. In comparison to models trained without noise, we observed that adding noise to the learned policy increases robustness of the learned policies and allowing the agent to be more stable against state fluctuations and model uncertainties. In addition, we evaluate the performance of a optimized reward function. Our results show that compared to previous design based on only the system state error, the new reward function achieves better drug control accuracy and therapeutic performance, thereby facilitating better overall control performance.

As shown in the Table 3, DDPG has demonstrated superior performance in tumor suppression and has also exhibited favorable efficacy in promoting the recovery of normal cells. This indicates that DDPG has enabled precise regulation of drug administration within complex biological systems, thereby providing a feasible technical pathway toward low-toxicity, high-efficiency personalized chemotherapy. Future work could further extend to modeling multi-drug synergistic effects, dynamically simulating tumor resistance mechanisms, and implementing closed-loop optimization strategies integrated with real clinical data–advancing intelligent therapeutic systems from theoretical research toward clinical translation.

**Table 3:** Comparison of Normal and Tumor Cell Levels and Cumulative Drug Dosage Across Different Treatment Protocols.

	Final proportion of normal cells	Final proportion of tumor cells	Total Drug Dose
Constant Drug (0.2)	0.9540	0.0247	160.00
DDPG (With Noise)	0.9976	0.0041	94.85
